# Transcutaneous Auricular Vagus Nerve Stimulation Alleviates Oxidative Stress and Improves Neurological Outcomes After Traumatic Brain Injury in Mice

**DOI:** 10.1002/cns.70913

**Published:** 2026-05-04

**Authors:** Xiaoxuan Li, Yifan Fu, Minghao Xu, Xinyu Xu, Jiuyu Gao, Shilin Liu, Zhige Wang, Chuandong Cheng, Tao Jiang

**Affiliations:** ^1^ Department of Neurosurgery The First Affiliated Hospital of Anhui Medical University Hefei Anhui China; ^2^ Anhui Public Health Clinical Center Hefei Anhui China; ^3^ First School of Clinical Medicine Anhui Medical University Hefei Anhui China; ^4^ Department of Neurosurgery, the First Affiliated Hospital of USTC, Division of Life Sciences and Medicine University of Science and Technology of China Hefei Anhui China

**Keywords:** neural repair, Nrf‐2/HO‐1 pathway, oxidative stress, transcutaneous auricular vagus nerve stimulation, traumatic brain injury

## Abstract

**Objective:**

Traumatic brain injury (TBI) can trigger secondary oxidative stress and exacerbate neurological dysfunction. This study investigated whether transcutaneous auricular vagus nerve stimulation (taVNS) attenuates oxidative stress and improves neurological recovery after TBI in mice.

**Methods:**

The TBI model was established using a controlled cortical impact (CCI) model. Seventy C57BL/6 mice were divided into the sham group, TBI group, taVNS group, MDHB (pathway agonist, administered alone) group, and ML385 (pathway inhibitor, administered 30 min before each taVNS session) group. Outcomes were assessed by Western blotting, ELISA, immunofluorescence, T2‐weighted MRI–based volumetric quantification of edema, and behavioral tests.

**Results:**

TBI markedly increased oxidative stress and neurological impairment, as reflected by reduced serum glutathione (GSH) and superoxide dismutase (SOD) levels, increased lesion and edema burden on MRI, and worsened behavioral performance. TaVNS significantly alleviated these changes, increasing GSH and SOD levels, reducing edema burden, and improving mNSS and open‐field performance. In addition, taVNS was associated with increased expression of antioxidant‐related proteins, including Nrf‐2 and HO‐1, whereas pharmacological inhibition with ML385 attenuated these beneficial effects.

**Conclusion:**

These findings suggest that taVNS may alleviate oxidative stress and improve neurological outcomes after TBI in mice, with possible involvement of the Nrf‐2/HO‐1 pathway.

## Graphical abstract

In a mouse model of traumatic brain injury, transcutaneous auricular vagus nerve stimulation (taVNS) attenuated oxidative stress and improved neurological recovery. taVNS enhanced Nrf‐2/HO‐1–related antioxidant responses, improved behavioral performance, and promoted edema resolution on MRI. Pharmacological modulation further supported the involvement of the Nrf‐2/HO‐1 pathway in these protective effects.

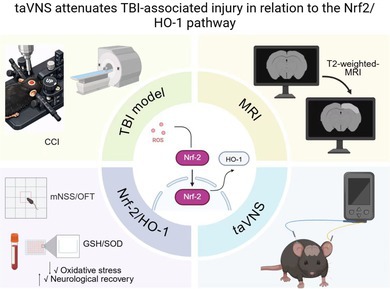

## 1 Introduction

**FIGURE 1:**
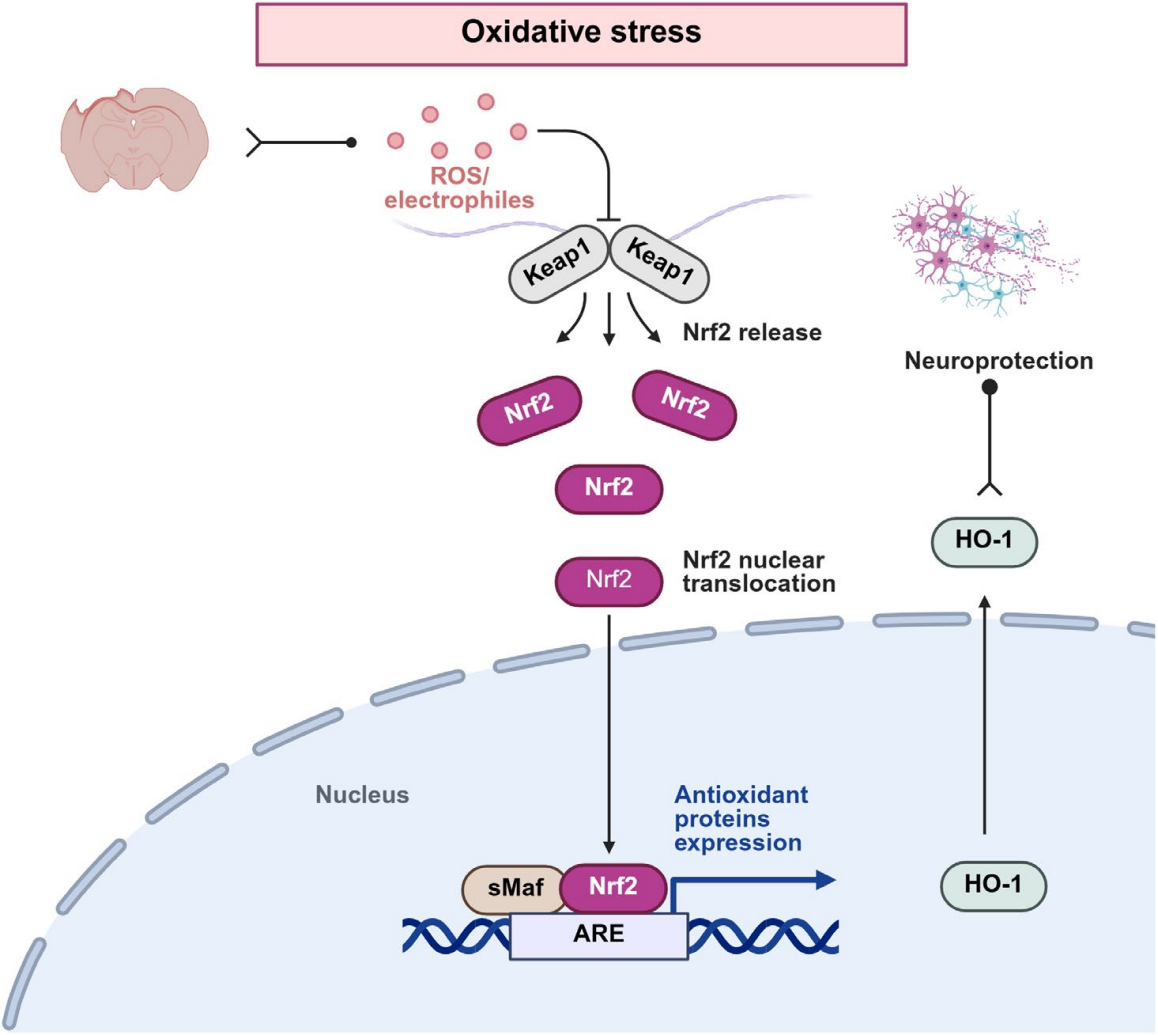
Schematic overview of the canonical Nrf‐2/HO‐1 antioxidant pathway in the context of oxidative stress. TBI induces oxidative stress and generates ROS that bind to Keap1, leading to Nrf‐2 release and nuclear translocation. Nrf‐2 in the nucleus binds to sMaf, which recognizes and binds to the antioxidant response element, which in turn induces the expression of antioxidant proteins HO‐1 and ultimately achieves neuroprotection.

Traumatic brain injury (TBI) is an important global health challenge that can cause death and significant disability, with millions of new cases reported annually [1]. TBI can be divided into primary and secondary injuries, where the primary injury is caused by mechanical damage and usually results in structural brain abnormalities and vascular dysfunction. Tissue necrosis and intracranial hemorrhage are common complications of TBI. Secondary injury includes a complex cascade of cell death, edema, oxidative stress, iron accumulation, endoplasmic reticulum stress, inflammation, and immune response [2]. Oxidative stress can upregulate the levels of free radicals, including reactive nitrogen species (RNS) and reactive oxygen species (ROS). After TBI, the electron transport chain (ETC) response to damaged cells enhances ROS production, and excessive ROS production impairs mitochondrial function, leading to mitochondrial membrane damage and lipid peroxidation [3]. Studies have shown that nuclear factor erythroid‐2‐related factor 2 (Nrf‐2) plays a considerable neuroprotective role in central nervous system (CNS) disorders [4]. Under normal physiological conditions, kelch‐like ECH‐related protein 1 (Keap1) binds Nrf‐2 and promotes its ubiquitination and proteasomal degradation [5]. In the presence of severe oxidative stress, Nrf‐2 dissociates from Keap1, translocates into the nucleus, and binds to the antioxidant response elements (AREs), thereby inducing the transcription of cytoprotective genes, including heme oxygenase‐1 (HO‐1), superoxide dismutase (SOD), and glutathione‐related enzymes (Figure 1) [6].

The vagus nerve is a complex neuroendocrine‐immunological network, and its wide distribution pattern emphasizes its important role in systemic regulation, information transfer, and immunomodulation. Novel non‐invasive (or percutaneous) VNS delivery systems rely on the cutaneous distribution of vagal fibers into the outer ear (vagal ear branch) and the neck (vagal cervical branch). The ear branch of the vagus nerve represents the tenth pair of cranial nerves in the skin (external auditory canal) [7]. After the activation of the ear branch of the vagus nerve, vagal afferent fibers can transmit nerve impulses to the nucleus of the solitary tract (NTS), nucleus of the locus coeruleus (LC), thalamus, hippocampus, amygdala, etc. [8] Transcutaneous auricular VNS (taVNS) can effectively treat a wide range of disorders, including depression, epilepsy, headache, tinnitus, atrial fibrillation, associative memory, schizophrenia, and pain [9]. taVNS resolves the need for surgical implantation, facilitating further research for a wide range of uses [10]. Previous animal experiments and clinical studies have indicated that taVNS may exert neuroprotective effects by modulating inflammation and oxidative stress, reducing brain edema, limiting excitotoxicity and apoptosis cascades, improving motor and cognitive functions, attenuating secondary brain injury, and promoting functional recovery [11].

A study investigating the therapeutic potential of taVNS in a mouse model of vitiligo indicated that vagal nerve stimulation enhances antioxidant capacity and attenuates apoptosis. These effects may be related to the neuromodulatory mechanisms that maintain redox homeostasis and apoptosis [12]. Similarly, VNS was shown to protect against oxidative damage and apoptosis in the liver in a rat model of I/R. Animal experiments suggest that VNS may protect against hepatic I/R injury by enhancing the Nrf‐2/HO‐1 signaling pathway [13]. These findings suggest that taVNS may be associated with Nrf‐2/HO‐1‐related antioxidant responses, but it remains unclear whether taVNS attenuates oxidative stress after TBI in association with this pathway. Therefore, the present study aimed to evaluate whether taVNS improves oxidative stress–related outcomes and neurological function after TBI, with evidence consistent with involvement of the Nrf‐2/HO‐1 pathway.

## 2 Materials and Methods

### 2.1 Animal Housing and Experimental Design

All procedures adhered to the ARRIVE 2.0 guidelines [14] and the National Institutes of Health Guide for the Care and Use of Laboratory Animals. All animal experiments were approved by the Animal Welfare and Ethics Committee of Anhui Medical University (LLSC20252156).

**FIGURE 2:**
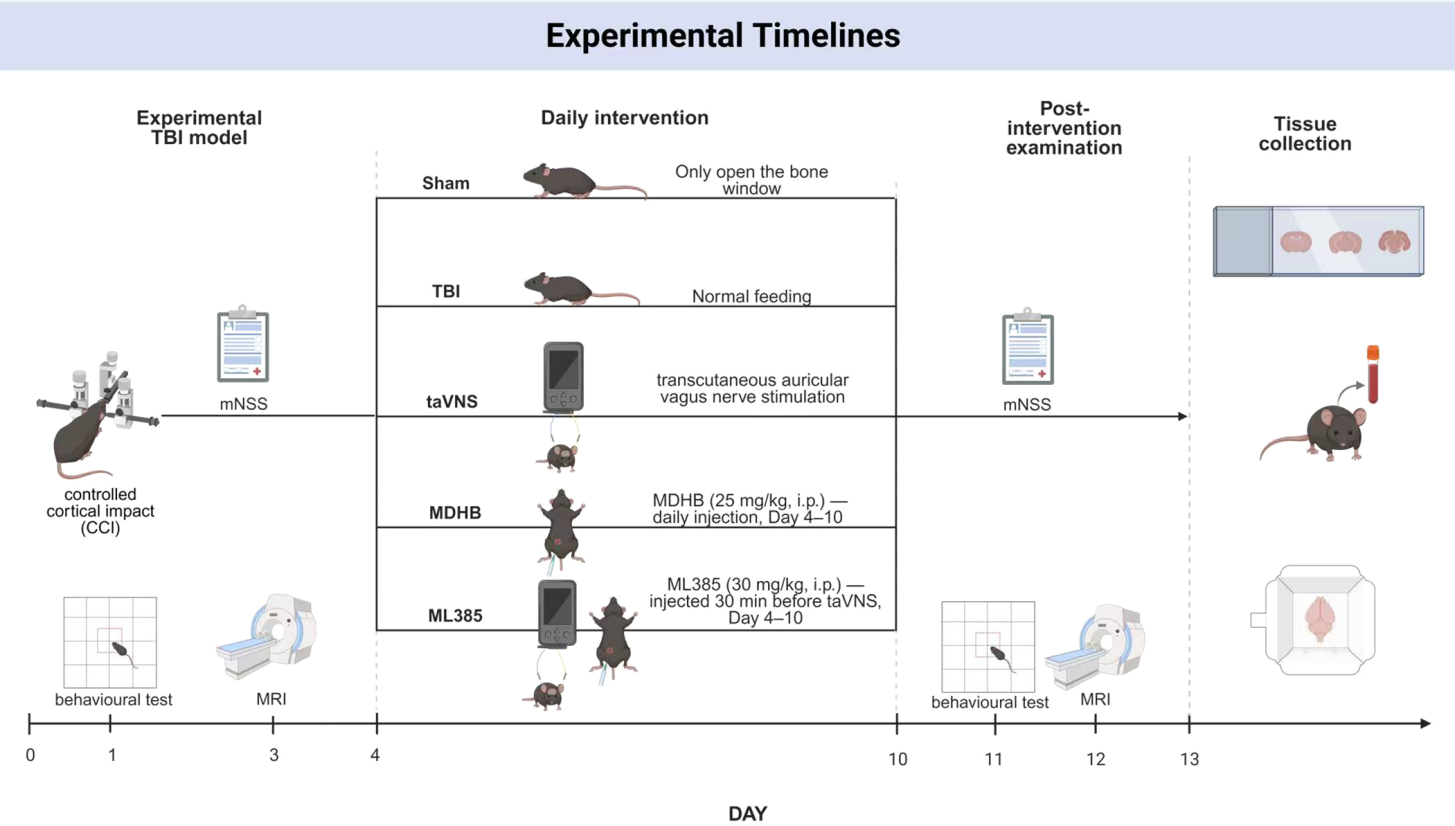
Experimental timeline. The experimental schedule was as follows: Day 0, controlled cortical impact (CCI) surgery and mNSS assessment; Day 1, behavioral testing; Day 3, T2‐weighted MRI; Days 4–10, interventions (taVNS group: Daily taVNS only; MDHB group: Daily intraperitoneal MDHB, 25 mg/kg, only; ML385 group: Intraperitoneal ML385, 30 mg/kg, administered 30 min before each taVNS session); Day 11, mNSS assessment and behavioral testing; Day 12, T2‐weighted MRI; Day 13, tissue collection for histological and biochemical analyses. CCI, controlled cortical impact; mNSS, modified neurological severity score; taVNS, transcutaneous auricular vagus nerve stimulation; TBI, traumatic brain injury.

In total, seventy C57BL/6 mice (10 weeks old) were purchased from Hefei Kisai Biotechnology Co. Ltd. Mice were acclimatized for 1 week under a 12‐h light/dark cycle with ad libitum access to food and water. Animals were randomly divided into five groups: sham group (*n* = 10), TBI group (*n* = 15), taVNS group (*n* = 15), MDHB group (*n* = 15, 25 mg/kg, i.p.), and ML385 group (*n* = 15, 30 mg/kg, i.p.). All groups underwent craniocerebral injury surgery except for the sham group, in which only the craniotomy was performed without impact. The group sizes reported here refer to the initial allocation; the final numbers analyzed for each outcome are provided in the corresponding figure legends. The experimental timeline is summarized in Figure 2.

### 2.2 Experimental Model of TBI

The mouse model of TBI was reproduced using a controlled cortical impact (CCI) device (YHCI99 Precision Craniocerebral Injury Impactor YHKJCI990313) according to previously published protocols [15]. Anesthesia was induced with 4% isoflurane in a mixed N_2_O/O_2_ (2:1) stream (0.5–1 L/min) for ~120 s and maintained with 1.5%–2.5% isoflurane [16]. After observing the gradual loss of the righting reflex, mice were positioned in a stereotaxic frame on a heating pad to maintain body temperature. Following scalp disinfection, a midline incision was made to expose the skull. A 3.5‐mm craniotomy was created over the right hemisphere (2.0 mm posterior to bregma and 2.0 mm lateral to the midline) to expose the intact dura. Moderate TBI was induced using a 3‐mm flat impactor tip (velocity 4.5 m/s, depth 2.0 mm, dwell time 200 ms). After impact, the incision was sutured, mice were kept warm during recovery, and a prophylactic antibiotic was administered intraperitoneally. Animals were returned to their home cages after recovery from anesthesia. Sham mice underwent identical procedures including craniotomy but without impact.

### 2.3 Modified Neurological Severity Score (mNSS)

At 10 am on days 0 and 11 of CCI, five areas of walking, sensory, tail lifting, balance, and reflex deficits were blindly scored by two trained researchers who were unaware of the reference to the experimental group. The lowest total score of 0 indicated the normal state without any neurological deficits. The highest score of 18 indicated unconsciousness or death.

### 2.4 Neurobehavioral Tests

Open‐field experiments on days 1 and 11 post‐TBI assessed mice's activity and exploration. A 40 × 40 × 30 cm open‐field apparatus was used, and the field was divided into a central zone and a peripheral zone. Mouse movement was recorded for 5 min using video‐tracking software under uniform illumination. The arena was cleaned with 75% ethanol between trials to minimize olfactory cues. Total distance traveled was used as an index of locomotor activity, and distance traveled in the center zone was used as an index of exploratory behavior or anxiety‐like behavior.

### 2.5 MRI Scanning Protocols

MRI scanning was conducted using a 9.4 T Bruker Biospec small animal scanner. A 20‐mm dedicated transmit/receive 1H mouse brain volume coil was used to conduct animal MRI, which offers satisfactory signal sensitivity and ease of animal setup. In this experiment, we scanned five groups of mice at two different time points (Day 3 and day 12 after TBI). Anesthesia was induced with 4% isoflurane and maintained at 1.5% during scanning. Body temperature was maintained at 37°C ± 1°C using a warm‐water circulation system. Respiration was monitored using an air cushion, and core temperature was monitored using a rectal probe [17]. T2‐weighted images were acquired with TR = 1500 ms and TE = 15 ms. The voxel resolution was 0.0481 × 0.0481 × 0.800 mm^3^, with a field of view (FOV) of approximately 20.0 × 20.0 × 16.0 mm^3^. Lesion and edema regions were segmented using ITK‐SNAP by manual delineation on each consecutive slice covering the entire injury extent. Total edema volume was then computed by voxel‐wise integration across the full stack, yielding a 3D volumetric measurement. To quantify temporal changes, edema volume was measured at Day 3 and day 12 for each mouse, and Δvolume was calculated as Volume_day3 − Volume_day12. Segmentation was performed by an investigator blinded to group allocation, and a subset of datasets was independently reviewed/segmented by a second investigator to confirm consistency.

### 2.6 Transcutaneous Auricular Vagus Nerve Stimulation

To stabilize the electrodes on the mouse ears during stimulation, light anesthesia was administered and maintained using a benchtop animal anesthetic ventilator system. Isoflurane was delivered in oxygen (0.5–1 L/min), with ~3% for induction and 1%–2% for maintenance during taVNS sessions [18]. The taVNS parameters used in this study (0.5 mA, 30 Hz, 30 min per day for 7 days) were chosen based on previously published protocols demonstrating effective activation of the auricular vagus nerve in mice [19]. In the screening, different current intensities (0.3, 0.5, and 0.7 mA) and frequencies (15 and 30 Hz) were tested to balance tolerability and physiological responsiveness; 0.5 mA at 30 Hz provided stable stimulation without visible discomfort or auricular skin irritation and was therefore used in the formal experiments. Stimulating clip electrodes were placed on the bilateral auricles and connected to a vagus nerve stimulator (Hangzhou Yijian Technology, YJT1‐240710002). Stimulation consisted of 0.5 mA at 30 Hz for 30 min daily, with a duty cycle of 30 s on/4.5 min off, for 7 days. Stimulation was performed at the same time each day. Electrode contact was optimized by gently moistening the auricle with saline to improve conductivity and ensure stable coupling. Effective stimulation was visually confirmed by slight auricular vibration.

To control for handling, anesthesia exposure, and electrode placement, mice in the sham and TBI groups were also fitted with the same auricular clip electrodes for an identical duration, but no electrical current was delivered (sham stimulation). All groups underwent identical handling and anesthesia exposure during the stimulation period.

### 2.7 Elisa

At the end of the experiment, all C57BL/6 mice were deeply anesthetized with 3% isoflurane (0.5 L/min oxygen flow) until loss of the righting and pedal withdrawal reflexes and were euthanized by cervical dislocation in accordance with the AVMA Guidelines for the Euthanasia of Animals (2020) [20]. Blood samples were allowed to clot at room temperature for 30 min and then centrifuged for 10 min at 3000 rpm to obtain serum. Serum was stored at −80°C and thawed on ice before the analysis. ELISA kits were used to prepare standards, detection antibodies, and substrate solutions. The absorbance of each well was read at 450 nm using an enzyme counter, and the concentrations of GSH and SOD in serum samples were calculated based on the standard curve.

### 2.8 Western Blotting

After the experiment, fresh brain tissue was collected, rinsed in ice‐cold PBS, and homogenized in RIPA lysis buffer containing protease inhibitors. Lysates were incubated on ice and centrifuged to collect supernatants. Protein concentration was determined and equal amounts of protein were separated by SDS–PAGE, transferred to PVDF membranes, and incubated with primary antibodies followed by appropriate secondary antibodies. Signals were visualized and quantified by densitometry, and target protein expression was normalized to GAPDH. All Western blot analyses were performed using total protein lysates rather than nuclear fractions; therefore, GAPDH served as the loading control for whole‐cell lysates.

### 2.9 Immunofluorescence

After the experiment, mice brains were perfused with paraformaldehyde, removed, fixed, and stored at −80°C. Sections were treated, dried, fixed with 4% paraformaldehyde, washed, permeabilized, and blocked with 5% BSA or 10% goat serum. Primary antibodies against Nrf‐2 and HO‐1 were added and incubated overnight at 4°C. Samples were washed, treated with fluorescent secondary antibody, incubated with DAPI, and observed under a microscope. Fluorescence signals were quantified with ImageJ.

### 2.10 TMT‐Based Quantitative Proteomics (TMT‐LC–MS/MS)

The whole brain tissues of three mice from the sham group, TBI group, and taVNS group were rapidly collected after anesthesia. The samples were immediately snap‐frozen in liquid nitrogen and stored at −80°C. Total protein was extracted and quantified, then subjected to reductive alkylation and trypsin digestion. Peptides were labeled using a TMT 11‐plex kit, fractionated by high‐pH RP‐HPLC, and analyzed by LC–MS/MS on an EASY‐nLC 1200 system coupled to a Q Exactive HF‐X mass spectrometer. Peptides were separated on a C18 column using a 4%–35% acetonitrile gradient over 120 min, and data were acquired in DDA mode with the specified resolution and fragmentation settings.

### 2.11 Data Processing and Analysis

Data were analyzed using GraphPad Prism 9.0 (GraphPad Software, USA). ImageJ 1.53 (NIH, USA) was used for image processing. Quantitative data are presented as mean ± SEM unless otherwise specified; non‐normally distributed data are presented as median (interquartile range). Normality was assessed using the Shapiro–Wilk test and homogeneity of variances using the Brown–Forsythe test. For two‐group comparisons, an unpaired *t*‐test was used for normally distributed data and the Mann–Whitney *U* test for non‐normal data. For comparisons among multiple groups, one‐way ANOVA with Tukey's post hoc test (normal distribution) or the Kruskal–Wallis test with Dunn's post hoc test (non‐normal distribution) was applied. For outcomes measured at multiple time points (mNSS and MRI lesion/edema volumes), a two‐way repeated‐measures ANOVA (group × time) or a mixed‐effects model was used as appropriate, followed by multiple‐comparisons testing. Correlations were analyzed using Pearson (normal data) or Spearman (non‐normal data) tests. All tests were two‐sided, and *p* < 0.05 was considered statistically significant. For proteomics, differentially expressed proteins were defined by |fold change| ≥ 1.5 with Benjamini–Hochberg corrected *p* < 0.05. KEGG annotation and enrichment were performed, with FDR < 0.05 considered significant; heatmaps were generated using the R package pheatmap (v1.0.12).

## 3 Results

### 3.1 Grouping of Experimental Mice

Seventy mice were initially enrolled, and 60 survived to the end of the study (Table 1). Most deaths occurred within 48 h after TBI, consistent with the known acute injury window. Mortality varied across groups, with the highest in ML385 (5/15), followed by TBI (3/15) and MDHB (2/15). No deaths occurred in the Sham or taVNS groups. Although attrition slightly reduced sample sizes, the final numbers remained within the acceptable range for behavioral and MRI analyses in TBI studies. Potential selection bias introduced by differential mortality is discussed in the Discussion section.

**TABLE 1:** Animal survival outcomes across experimental groups.

Group	Expected number	Actual number of survivors	Number of deaths
Sham group	10	10	0
TBI group	15	12	3
taVNS Group	15	15	0
MDHB Group	15	13	2
ML385 Group	15	10	5
Total	70	60	10

Abbreviations: taVNS, transcutaneous auricular vagus nerve stimulation; TBI, traumatic brain injury.

The initial group sizes are reported in the Experimental design section. Because different outcome measures require different sample preparation and quality‐control criteria, the numbers of animals analyzed (*n*) may differ across assays (MRI, Western blotting, ELISA, and immunofluorescence). For each assay, n represents the number of mice (biological replicates) included in the final analysis, and the exact n for each panel is provided in the corresponding figure legend. When multiple fields were analyzed per mouse (immunofluorescence), values were first averaged within each animal, and statistical comparisons were performed using the animal as the experimental unit. Data were excluded only based on pre‐defined technical criteria (excessive MRI motion artifacts, insufficient sample quantity, hemolyzed serum, or failed staining), and not based on the study outcomes.

### 3.2 Proteomic Profiling Suggested That taVNS Modulated TBI‐Related Molecular Alterations

**FIGURE 3:**
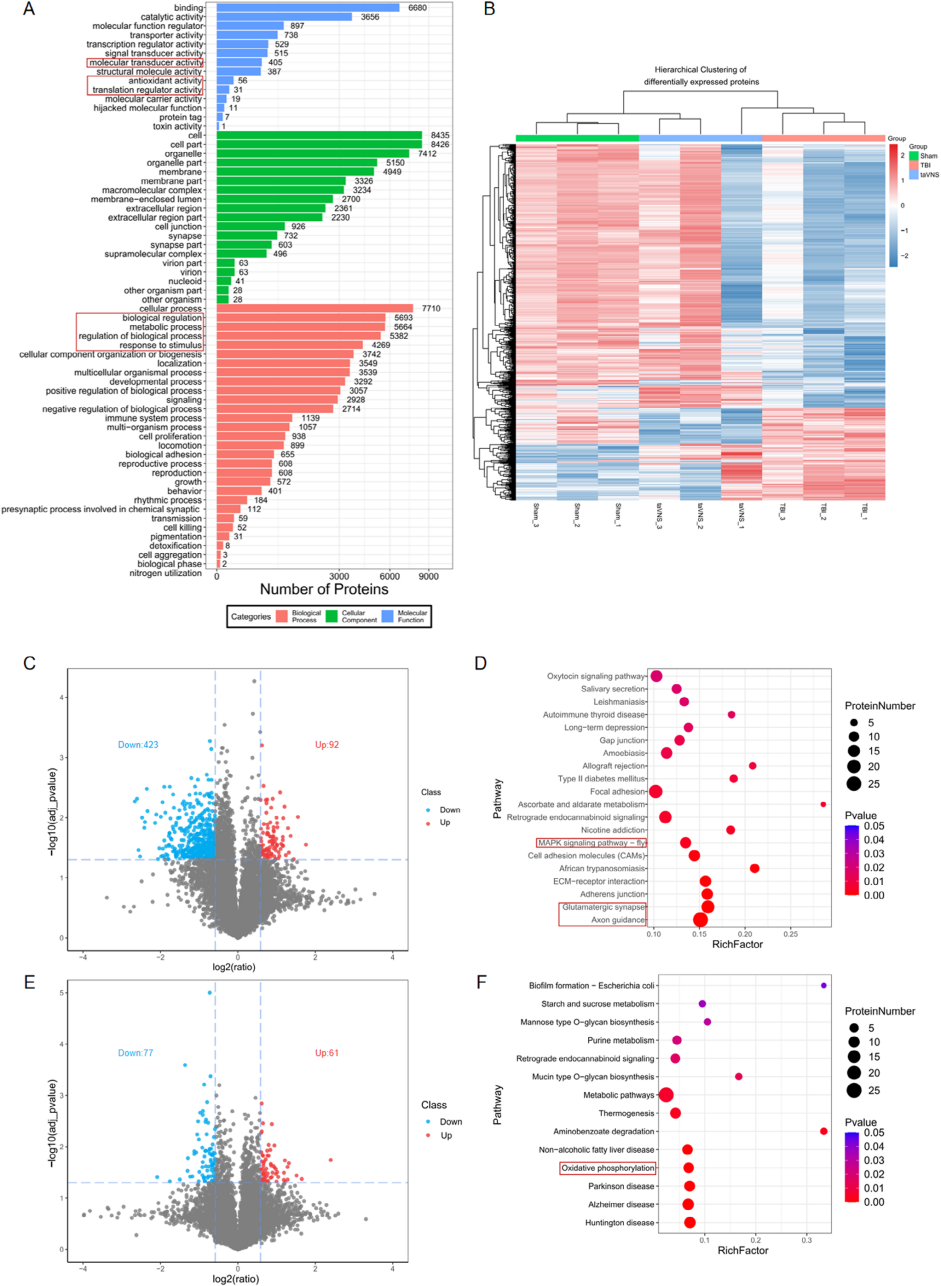
Results of proteomics analysis. (A) GO enrichment analysis: GO_MF_term, molecular function of GO analysis. GO_BP_term, biological process of GO analysis. (B) The heatmap illustrates the differentially expressed proteins identified when comparing the sham, TBI and taVNS groups. The figure illustrates a hierarchical cluster analysis. The color scale represents log2‐transformed TMT reporter ion intensities after normalization. Each row corresponds to a protein. Red indicates high expression levels, blue indicates low expression levels and white indicates medium expression levels. (C) Volcano plots showing the distribution of differential proteins in TBI versus Sham; (D) KEGG enrichment analyses showing the signaling pathways involved in the differential proteins in TBI versus Sham; (E) Volcano plots showing the distribution of differential proteins in taVNS vs. TBI; (F) KEGG enrichment analyses showing the signaling pathways involved in the differential proteins in taVNS versus TBI. Sham: *N* = 3; TBI: *N* = 3; taVNS: N = 3. n indicates the number of mice included in each analysis; taVNS, transcutaneous auricular vagus nerve stimulation; TBI, traumatic brain injury.

GO functional annotation analysis revealed that three core ontologies exhibited significant enrichment patterns in the proteomics profiles of the Sham, TBI, and taVNS groups: molecular function (MF), cellular component (CC), and biological process (BP). Within the molecular function category, ‘antioxidant activity’ was found to be significantly enriched, directly involving proteins involved in redox homeostasis and oxidative stress responses, which form the core focus of this study. Furthermore, ‘transcription factor activity’ and ‘molecular function regulators’ have been demonstrated to be associated with oxidative stress. The biological process ontology further corroborated this finding, with ‘response to stimuli’, ‘biological processes and biological process conditions’, and ‘metabolic processes’ all demonstrating significant enrichment. Collectively, these BP terms indicate the widespread activation of stress adaptation pathways in TBI, and taVNS intervention may regulate these responses (Figure 3A).

The hierarchical clustering heatmap visualized the expression patterns of the differential proteins in the two groups of TBI/sham and taVNS/TBI (Figure 3B). TBI‐induced differential proteins showed a clear trend of reverse regulation after taVNS. This reversed expression pattern suggests that taVNS may systematically correct TBI‐induced oxidative stress imbalance and cellular dysfunction by remodeling the expression pattern of differential proteins.

The volcano plot of differential proteins in the TBI/Sham group showed that 515 significantly different proteins (fold change ≥ 1.5, BH‐adjusted *p* < 0.05) in the TBI group compared to the sham group (Figure 3C). Among them, upregulated proteins (in blue) were bidirectionally distributed with downregulated proteins (in red). KEGG pathway enrichment analysis also revealed that TBI significantly perturbed neural function‐related pathways, such as axon guidance, glutamatergic synapse, MAPK signaling pathway, etc. (Figure 3D). Abnormal activation or inhibition of these pathways indicates extensive disturbances in the structural integrity and signaling network of the nervous system after TBI. Volcano plots of the taVNS/TBI group showed significantly different protein populations (fold change ≥ 1.5, BH‐adjusted *p* < 0.05). After taVNS, the expression trend of some of the proteins became similar to the sham group (Figure 3E). In KEGG pathway enrichment analysis, taVNS was significantly enriched for oxidative phosphorylation and other pathways related to energy metabolism and antioxidant defense (Figure 3F). The enrichment of the oxidative phosphorylation pathway suggested that taVNS may ameliorate energy metabolism disorder after TBI by regulating mitochondrial function, while enrichment of antioxidant‐related pathways was consistent with altered redox regulation after taVNS and may reflect antioxidant‐related molecular responses. These findings suggest that taVNS may partially reverse TBI‐related molecular disturbances.

### 3.3 CCI Led to Neurological Deficits and Oxidative Stress

**FIGURE 4:**
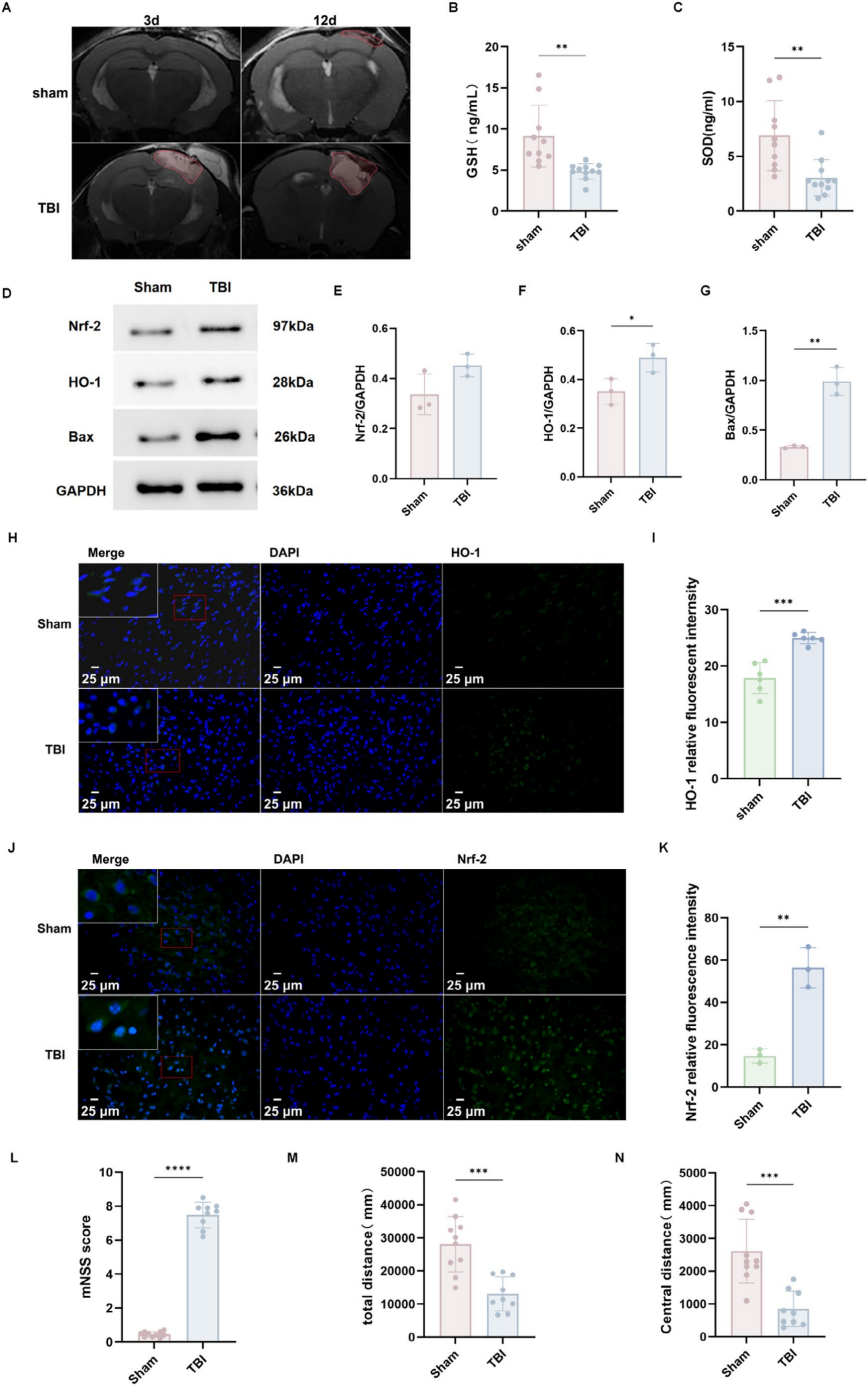
TBI induces oxidative stress and neurological deficits. (A) Representative T2‐weighted MRI images of the brain in Sham and TBI groups at 3 and 12 days post‐injury. Sham: *N* = 8; TBI: *N* = 12. (B, C) Serum levels of GSH and SOD. Sham: *N* = 10; TBI: *N* = 12. (D) Western blot analysis of Nrf‐2, HO‐1, and Bax protein expression. Sham: *N* = 3; TBI: *N* = 3. (E‐G) Quantitative analysis of HO‐1/GAPDH, Nrf‐2/GAPDH, and Bax/GAPDH ratios. (H–K) Immunofluorescence staining and quantitative analysis of HO‐1 and Nrf‐2 expression. HO‐1: Sham: *N* = 6; TBI: *N* = 6; Nrf‐2: Sham: *N* = 3; TBI: *N* = 3. (L–N) mNSS and locomotor activity (total distance and center distance) in the open field test. Sham: *N* = 10; TBI: *N* = 9. Data are presented as mean ± SEM. n indicates the number of mice included in each analysis; for immunofluorescence, multiple fields were averaged per mouse. *p < 0.05, **p < 0.01, ***p < 0.001, ****p < 0.0001. taVNS, transcutaneous auricular vagus nerve stimulation; TBI, traumatic brain injury.

Three days after CCI modeling, T2‐weighted MRI confirmed robust lesion formation and cerebral edema, supporting successful establishment of the CCI model (Figure 4A). Consistent with oxidative stress, serum GSH levels were reduced in TBI mice compared with Sham (*p* < 0.01; Figure 4B), and serum SOD activity was also decreased (*p* < 0.01; Figure 4C). Western blotting showed increased HO‐1 (*p* < 0.05) and Bax (p < 0.01) expression after TBI, while total Nrf‐2 showed only a modest, non‐significant increase (Figure 4D–G), suggesting partial activation of endogenous antioxidant defenses. Immunofluorescence further showed increased Nrf‐2 and HO‐1 immunoreactivity in peri‐lesional regions (Figure 4H–K). Behaviorally, TBI mice exhibited higher mNSS scores (*p* < 0.001) and reduced locomotor activity (*p* < 0.01) and center distance (*p* < 0.001) in the open‐field test, consistent with neurological impairment and increased anxiety‐like behavior (Figure 4L–N).

### 3.4 taVNS Exerted Neuroprotective and Antioxidant Effects After CCI

**FIGURE 5:**
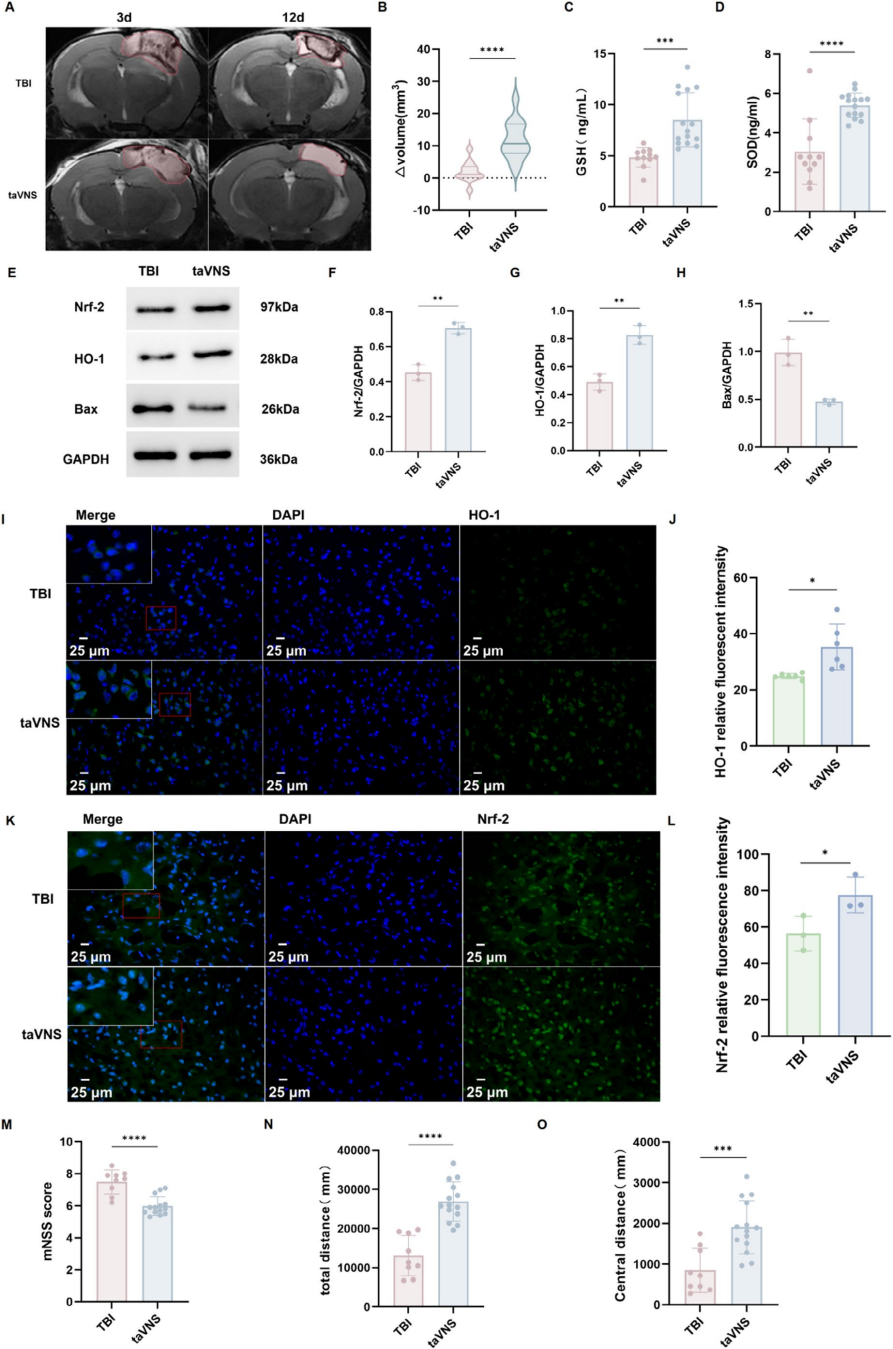
Effects of taVNS on neurological function and oxidative stress after TBI. (A) Representative T2‐weighted MRI images of the brain in TBI and taVNS groups at 3 and 12 days post‐injury. (B) Change in brain edema volume between Day 3 and day 12 after TBI (Δvolume = volume Day3 − volume Day12). TBI: *N* = 12; taVNS: *N* = 13. (C, D) Serum levels of GSH and SOD. TBI: *N* = 11; taVNS: *N* = 15. (E) Western blot analysis of Nrf‐2, HO‐1, and Bax protein expression. TBI: *N* = 3; taVNS: *N* = 3. (F–H) Quantitative analysis of HO‐1/GAPDH, Nrf‐2/GAPDH, and Bax/GAPDH ratios. (I–L) Immunofluorescence staining and quantitative analysis of HO‐1 and Nrf‐2 expression. HO‐1: TBI: *N* = 6; taVNS: *N* = 6; Nrf‐2: TBI: *N* = 3; taVNS: *N* = 3. (M–O) Modified neurological severity score (mNSS) and locomotor activity (total distance and center distance) in the open field test. TBI: *N* = 9; taVNS: *N* = 14. Data are presented as mean ± SEM. n indicates the number of mice included in each analysis; for immunofluorescence, multiple fields were averaged per mouse. *p < 0.05, **p < 0.01, ***p < 0.001, ****p < 0.0001. taVNS, transcutaneous auricular vagus nerve stimulation; TBI, traumatic brain injury.

TaVNS was administered from days 4–10 after TBI. Compared with untreated TBI mice, taVNS improved edema resolution on T2‐weighted MRI. Δvolume (day 3 − day 12) was larger in the taVNS group, indicating a greater reduction over time (*p* < 0.0001, Figure 5A,B). Biochemically, we measured serum GSH (Figure 5C) and serum SOD (Figure 5D) levels in the taVNS and TBI groups to investigate the effect of taVNS on oxidative stress in mice with TBI. The results showed levels were increased by taVNS(GSH: *p* < 0.001; SOD: *p* < 0.001). Additionally, Western blotting (Figure 5E) showed higher Nrf‐2 (Figure 5F) and HO‐1 (Figure 5G) levels (*p* < 0.01) and lower Bax levels in taVNS‐treated mice (p < 0.01, Figure 5H). Furthermore, Immunofluorescence demonstrated increased Nrf‐2 and HO‐1 immunoreactivity in the taVNS group (*p* < 0.05, Figure 5I–L). To investigate the effect of taVNS on the recovery of neural function, we compared taVNS with the TBI group and found that mNSS scores were reduced (*p* < 0.0001, Figure 5M), indicating improved neurological function. In the open field test, we observed that mice undergoing taVNS exhibited increased locomotor activity (*p* < 0.0001) and center distance (*p* < 0.0001, Figure 5N,O).

### 3.5 Pharmacological Modulation of Nrf‐2/HO‐1 Signaling Altered taVNS‐Associated Antioxidant and Neurobehavioral Outcomes

**FIGURE 6:**
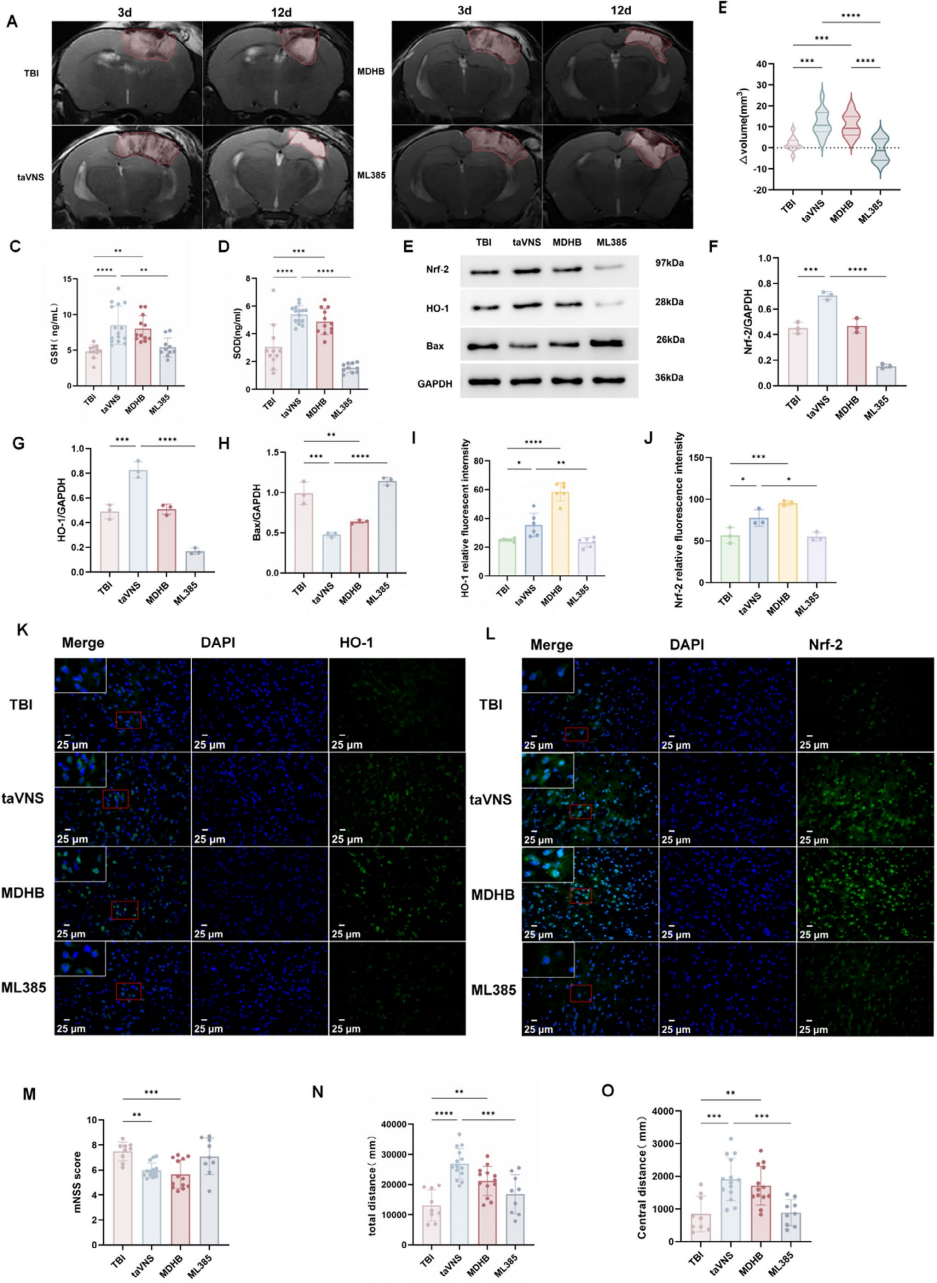
Pharmacological modulation of Nrf‐2 signaling in the context of taVNS. (A) Representative T2‐weighted MRI images of the brain in TBI, taVNS, MDHB (Nrf‐2 agonist), and ML385 (Nrf‐2 inhibitor) groups at 3 and 12 days post‐injury. (B) Change in brain edema volume between Day 3 and Day 12 after TBI (Δvolume = volume Day3 − volume day12). TBI: *N* = 12; taVNS: *N* = 12; MDHB: *N* = 12; ML385: *N* = 10. (C, D) Serum levels of GSH and SOD. TBI: *N* = 11; taVNS: *N* = 14; MDHB: *N* = 12; ML385: *N* = 10. (E) Western blot analysis of Nrf‐2, HO‐1, and Bax protein expression. TBI: *N* = 3; taVNS: *N* = 3; MDHB: *N* = 3; ML385: *N* = 3. (F–H) Quantitative analysis of HO‐1/GAPDH, Nrf‐2/GAPDH, and Bax/GAPDH ratios. (I–L) Immunofluorescence staining of HO‐1 and Nrf‐2. HO‐1:TBI: *N* = 6; taVNS: *N* = 6; MDHB: *N* = 6; ML385: N = 6; Nrf‐2:TBI: *N* = 3; taVNS: *N* = 3; MDHB: *N* = 3; ML385: N = 3. (M–O) mNSS and locomotor activity (total distance and center distance) in the open field test. TBI: *N* = 9; taVNS: *N* = 15; MDHB: *N* = 13; ML385: *N* = 9. Data are presented as mean ± SEM. n indicates the number of mice included in each analysis; for immunofluorescence, multiple fields were averaged per mouse. *p < 0.05, **p < 0.01, ***p < 0.001, ****p < 0.0001. taVNS, transcutaneous auricular vagus nerve stimulation; TBI, traumatic brain injury.

To further probe the involvement of Nrf‐2/HO‐1 signaling, we compared taVNS with pharmacological activation (MDHB) and inhibition (ML385). The MDHB group only received an intraperitoneal injection of MDHB (an Nrf‐2/HO‐1 pathway agonist) following TBI, while the ML385 group received an intraperitoneal injection of ML385 (an Nrf‐2/HO‐1 pathway inhibitor) 30 min prior to each taVNS stimulation. On MRI, taVNS and MDHB produced a trend toward improved edema resolution relative to TBI (taVNS vs. TBI: *p* < 0.0001; MDHB vs. TBI: *p* < 0.0001), whereas ML385 attenuated the edema‐reducing effect of taVNS (*p* < 0.0001) (Figure 6A,B). Serum GSH (Figure 6C) and SOD (Figure 6D) levels were increased in the taVNS and MDHB groups and were reduced by ML385 compared with taVNS. Western blotting and immunofluorescence also confirmed higher Nrf‐2 and HO‐1 levels in taVNS‐treated mice, with similar but not always statistically significant trends in the MDHB group; ML385 reduced these taVNS‐associated increases (Figure 6E–L). Additionally, Bax levels were decreased by taVNS and showed a similar trend with MDHB, while ML385 blunted these changes (Figure 6E–H). Behaviorally, both taVNS and MDHB improved mNSS scores and open‐field performance, whereas ML385 diminished these improvements (Figure 6M–O). Overall, these findings provide pharmacological evidence consistent with participation of Nrf‐2/HO‐1 signaling in taVNS‐associated antioxidant and neurobehavioral benefits.

## 4 Discussion

TBI is a major cause of death and long‐term disability and is characterized by a cascade of secondary injuries in which oxidative stress plays a central role. In the present study, we evaluated whether taVNS mitigates oxidative stress–related injury and improves neurological outcomes in a mouse CCI model. Across imaging, biochemical, and behavioral endpoints, taVNS was associated with reduced lesion/edema burden, improved functional performance, and enhanced antioxidant capacity. At the molecular level, taVNS was accompanied by changes in Nrf‐2/HO‐1‐related signaling, which may be associated with its antioxidant effects after TBI. Firstly, comparative analyses between the TBI and sham groups revealed pronounced changes in the proteomic landscape after TBI. The volcano plots showed a broad distribution of upregulated and downregulated proteins, suggesting that TBI is accompanied by widespread molecular disturbances, consistent with the engagement of multiple secondary injury processes, including oxidative stress–related responses, apoptosis‐related signaling, and neuroinflammatory cascades [21]. Notably, pathway enrichment in TBI/Sham comparison highlighted glutamatergic synapse and axon guidance pathways. Abnormal metabolism of glutamate as a major excitatory neurotransmitter in the brain leads to excitotoxicity, and abnormality of the axon guidance pathway may affect the repair and remodeling of neural circuits. Studies have shown that abnormal function of glutamate transporter triggered by transmitter accumulation and impaired neuroplasticity can lead to secondary brain injury after TBI, which also provides a theoretical support for the disorders of neurotransmitter system‐related pathways in the present study [22]. Secondly, in the taVNS/TBI group, taVNS was associated with a partial normalization of the TBI‐altered proteomic profile. In the volcano plot of the taVNS/TBI group, we observed that the distribution of differential proteins was different from that in the TBI/Sham group, suggesting that taVNS may modulate a subset of proteins and pathways perturbed after injury. In the hierarchical clustering heatmap (TBI‐vs‐Sham‐taVNS‐vs‐TBI) the taVNS/TBI samples exhibited a protein‐expression pattern closer to the sham group than to the untreated TBI group, indicating a remodeling trend in the post‐injury proteomic profile. KEGG enrichment of oxidative phosphorylation in the taVNS/TBI comparison may reflect changes related to mitochondrial bioenergetics, which is closely linked to oxidative stress after TBI [23]. Meanwhile, the present study reported the enrichment of the chemical carcinogenesis‐reactive oxygen species pathway in the taVNS/TBI group, suggesting that taVNS may influence redox‐related processes regulating the balance between ROS generation and scavenging. Functional analyses indicated that taVNS may indirectly affect oxidative stress in mice with TBI by regulating other pathways associated with oxidative stress. The results of the present study are consistent with those of previous reports on the neuroprotective effects of VNS. For instance, previously, it was found that VNS can attenuate inflammatory responses by inhibiting the nuclear factor‐κB (NF‐κB) pathway and suppress oxidative stress by upregulating the activity of antioxidant enzymes, such as superoxide dismutase (SOD) [24]. These findings further support a protective role of VNS in neural injury and are broadly consistent with previous observations in TBI.

In addition to the regulatory mechanisms of oxidative stress‐related pathways at the proteomic level, there is a close association between TBI‐induced abnormal protein expression and neurological dysfunction. Typically, TBI can lead to neuronal loss, tissue necrosis, and vascular disruption, which contribute to lesion formation and cerebral edema. In our study, T2‐weighted MRI showed evident injury‐related hyperintensity and edema at day 3 after trauma, and residual edema signals remained detectable at day 12. Moreover, severe oxidative stress after TBI is an important mechanism underlying neuronal damage. Previous studies [25, 26] detailed the involvement of oxidative stress in secondary injury after TBI, and also reported that mitochondrial dysfunction after TBI is closely associated with oxidative stress. Meanwhile, the serum levels of GSH and SOD were reduced in TBI mice. The Nrf‐2 pathway is a central regulator of cellular antioxidant responses [27, 28]. Nrf‐2 dissociates from Keap‐1 and then enters the nucleus, where it binds to the antioxidant response element (ARE) and activates the expression of a series of antioxidant enzymes and detoxification enzymes. These enzymes scavenge excessive ROS, maintain intracellular redox homeostasis, and mitigate the cellular damage caused by oxidative stress. Huang et al. [29] revealed that Nrf‐2 promotes cellular antioxidant defense, suppresses the inflammatory response, and inhibits apoptosis, thereby playing an important role in neuroprotection after TBI. Enhancing cellular antioxidant capacity attenuates oxidative stress and promotes neurological recovery. Increased expression of Nrf‐2 and HO‐1 proteins in brain tissues after TBI suggests the activation of the endogenous antioxidant defense system. Due to vascular damage, glial scarring formed by activated astrocytes, and limited brain tissue regeneration [30], TBI severely impairs neurological functions, such as motor and cognitive functions. In this study, mice exhibited obvious neurological deficits after TBI, with significantly higher mNSS scores. Open‐field experiments showed reduced activity and increased anxiety‐like behaviors, suggesting serious neuromotor dysfunctions after TBI.

Vagus nerve stimulation, as a neuromodulatory technology, can exert neuroprotective effects by modulating neurotransmitters and neural networks. Wang et al. [7] outlined the potential of VNS in treating neurological disorders, such as epilepsy, depression, Alzheimer's disease (AD), TBI, etc. Aniwattanapong and Follesa [31, 32] investigated the effects of VNS on memory and neuroplasticity. Mahoney et al. [33] discussed neuromodulation approaches, including VNS‐related strategies, in neuropsychiatric disorders. Previous reviews have summarized the preclinical and clinical evidence supporting VNS in TBI [11]. Zhang [34] emphasized the non‐invasive advantages of percutaneous VNS to improve neurological recovery and suppress neuroinflammation and oxidative stress. Moreover, the present study indicated that taVNS was associated with improved injury‐related outcomes after TBI. After TBI, T2‐weighted MRI showed that taVNS reduced the volume of brain injury and the volume of cerebral edema, which is consistent with that reported by previous studies [11, 35]. Additionally, we found elevated serum levels of GSH and SOD, increased protein expression of Nrf‐2 and HO‐1 in the brain tissue, and decreased expression levels of Bax. Immunofluorescence with DAPI counterstaining suggested more prominent Nrf‐2 immunoreactivity after taVNS. Previous studies have shown that the Nrf‐2/HO‐1 pathway possesses antioxidant and anti‐inflammatory effects in various diseases. For instance, Deng [36] found that VNS protects against lung injury induced by hepatic ischemia. To further probe pathway involvement, we employed bidirectional pharmacological modulation. MDHB, as an agonist of the Nrf‐2/HO‐1 pathway, produced protective trends resembling those observed with taVNS, including enhanced antioxidant readouts and reduced edema‐related MRI measures. Conversely, ML385, a selective Nrf‐2 inhibitor administered prior to taVNS sessions, attenuated several taVNS‐associated benefits. Taken together, these results provide supportive pharmacological evidence that Nrf‐2/HO‐1 signaling may contribute to taVNS‐associated protection, rather than definitive proof of direct pathway mediation. Nevertheless, pharmacological interventions can have off‐target effects and do not substitute for genetic loss‐of‐function approaches.

In previous studies, brain water content measurement has been the most widely used classical method for assessing brain edema in pathological states, such as TBI. This method usually involves the execution of animals at the end of the experiment and obtaining brain tissue samples. The percentage of water content is calculated by accurately measuring the difference between the wet weight of the tissue sample and the dry weight of the dried tissue sample using the following formula: (wet weight—dry weight)/wet weight × 100% [37]. Although this method can provide objective numerical data, its limitations are more prominent. It is a destructive, ex vivo analysis, which cannot monitor the same subject in vivo, and it is difficult to track the real‐time changes in brain edema during disease progression or treatment. It can only reflect the average water content of the brain tissue as a whole, but it cannot accurately present the spatial distribution of edema in different regions of the brain. Such regional variations often exhibit different characteristics, closely associated with the localization of neurological impairment [17, 38]. In contrast, this study innovatively used magnetic resonance T2‐weighted imaging to assess brain edema. T2WI works based on the differences in lateral relaxation times (T2 values) of water molecules in tissues. The enlargement of the extracellular gap in brain edema increases the freedom of movement of water molecules, which in turn prolongs the T2 value and manifests as a high‐signal region on imaging [38, 39]. This enables non‐invasive, longitudinal assessment in vivo, allowing repeated measurements and visualization of lesion/edema morphology and spatial distribution [17]. In our study, we used longitudinal T2‐weighted MRI with 3D volumetric segmentation to quantify lesion/edema burden over time, enabling within‐animal comparison between day 3 and day 12. This approach supports dynamic evaluation of edema resolution and complements behavioral and molecular readouts. Consistent with this, taVNS reduced the MRI‐derived edema burden and facilitated edema resolution during the post‐injury period.

Despite the encouraging trends observed in the acute phase, our study was not designed to determine whether taVNS provides sustained benefit beyond the early post‐injury window. T2‐weighted MRI readouts (edema volume and edema burden) therefore primarily suggest an association with improved early injury evolution, and longer‐term follow‐up with serial imaging and behavioral testing will be needed to assess durability. Mechanistically, although our results together with bidirectional pharmacological modulation are consistent with involvement of Nrf‐2/HO‐1 signaling, the absence of genetic loss‐of‐function validation precludes conclusions regarding definitive pathway dependency. In addition, attenuation by ML385 indicates pathway participation but does not demonstrate that taVNS directly and specifically engages Nrf‐2/HO‐1 signaling as its primary mechanism of action. Because Nrf‐2/HO‐1 is a broadly inducible antioxidant response after TBI, the observed pathway changes may reflect either a contributing mechanism or a downstream adaptive response secondary to upstream taVNS‐mediated processes. Pharmacological effects may also partially reflect parallel redox‐regulatory circuits. Moreover, because nuclear–cytoplasmic fractionation was not performed, the immunofluorescence evidence should be considered supportive rather than conclusive for Nrf‐2 nuclear translocation. Future studies integrating genetic validation, expanded profiling of downstream Nrf‐2 targets (NQO1/GCLC/GCLM/SLC7A11/GST), and functional MRI network analyses will help clarify how molecular programs relate to circuit‐level recovery after TBI, in line with accumulating evidence that taVNS/VNS modulates inflammatory and oxidative stress responses across disease contexts [40, 41, 42, 43].

## 5 Conclusion

This study suggests that taVNS may alleviate oxidative stress and improve neurological outcomes after TBI in mice. The accompanying molecular and pharmacological findings support possible involvement of the Nrf‐2/HO‐1 pathway, but do not establish definitive causal mediation. These findings support further investigation of taVNS as a non‐invasive neuromodulatory strategy for TBI.

## Author Contributions

**Xiaoxuan Li:** conceptualization, methodology, investigation, formal analysis, writing – original draft; **Yifan Fu:** conceptualization, methodology, investigation, writing – original draft; **Minghao Xu:** investigation; **Xinyu Xu:** investigation; **Jiuyu Gao:** investigation; **Shilin Liu:** investigation, project administration; **Zhige Wang:** investigation; chuandong cheng: supervision, writing – review and editing; **Tao Jiang:** supervision, writing – review and editing. All authors made a significant contribution to the work reported, whether that is in the conception, study design, execution, acquisition of data, analysis and interpretation, or in all these areas; took part in drafting, revising or critically reviewing the article; gave final approval of the version to be published; have agreed on the journal to which the article has been submitted; and agree to be accountable for all aspects of the work.

## Funding

This research was supported by the Anhui Provincial Scientific Research Projects for Universities (2023AH040076) and the Anhui Provincial Traditional Chinese Medicine Inheritance and Innovation Research Project (2025CCCX014).

## Ethics Statement

The experiments were conducted strictly in accordance with the Guide for the Care and Use of Laboratory Animals (National Institutes of Health, USA). All animal experiments were approved by the Animal Welfare and Ethics Committee of Anhui Medical University (LLSC20252156).

## Conflicts of Interest

The authors declare no conflicts of interest.

## Data Availability

The data that support the findings of this study are available from the corresponding author (Tao Jiang) upon reasonable request.
